# Effectiveness of an online training program for improving nurses’ competencies in disaster risk management

**DOI:** 10.1186/s12912-023-01497-1

**Published:** 2023-09-27

**Authors:** Jamileh Farokhzadian, Hojjat Farahmandnia, Asghar Tavan, Gülcan Taskiran Eskici, Faezeh Soltani Goki

**Affiliations:** 1 Nursing Research Center, Kerman University of Medical Sciences, Kerman, Iran; 2 Health in Disasters and Emergencies Research Center, Institute for Futures Studies in Health, Kerman University of Medical Sciences, Kerman, Iran; 3 Faculty of Management and Medical Information Sciences, Kerman University of Medical Sciences, Kerman, Iran; 4 Department of Nursing Administration, Faculty of Health Sciences, Ondokuz Mayis University, Samsun, Turkey; 5 Student Research Committee, Razi Faculty of Nursing and Midwifery, Kerman University of Medical Sciences, Kerman, Iran

**Keywords:** Incidents and disasters, Disaster risk management, Core competencies, Online training, Nurses

## Abstract

**Background:**

Nurses’ incompetency in disaster risk management can have many negative consequences during disasters, so it is important to prepare nurses and improve their competencies in disaster risk management. This study was conducted with the aim of investigating the effectiveness of an online training program to improve competencies in disaster risk management.

**Method:**

This interventional study was conducted on nurses working in a specialized trauma hospital affiliated with the Kerman University of Medical Sciences in southeastern Iran in 2023. Eighty-one nurses were randomly assigned into two interventions (n = 42) and control groups (n = 39). The intervention group received an online training program in four sessions, and both groups electronically completed the demographic questionnaire and the nurses’ perceptions of disaster core competencies scale (NPDCC) before and one month after the intervention.

**Results:**

The study results showed no significant difference in disaster competency scores between the two groups before the intervention (p < 0.51), but the NPDCC score in the intervention group was statistically significant after the intervention compared to before the intervention (p < 0.02) and no statistically significant difference was observed between the two groups after the intervention (p < 0.16).

**Conclusion:**

While the online training program was found to significantly improve the NPDCC score of nurses in the intervention group, this increase was not significant when compared to the control group. Therefore, we suggest continuous practical exercises and maneuvers to improve nurses’ perception of the competencies required for effective disaster management.

## Introduction

Disasters are natural or man-made incidents that influence the exposure of a community and require a capacity beyond existing facilities [1]. Disasters occur quickly and without prior warning resulting in economic, social, healthcare damage [2]. With the increase in disasters worldwide, attention has been paid to the role of healthcare providers in disaster management, as adverse situations caused by disasters can have catastrophic effects [3].

The healthcare system is primarily responsible for the impact on people’s health; therefore, hospitals play an important role in reducing the adverse effects of disasters [4].

The availability of human resources, equipment and processes is one of the important aspects of disaster risk management [5]. The role of nurses, as full-fledged partners of the healthcare team in the front line of disaster risk management, is very important [6]; they take responsibility for new practices, tasks, solutions, suggestions and decisions according to their knowledge, skills and competencies [7]. For example, nurses play a key role in mental and social health planning, training and counseling, coordinating care, providing disaster information and triage, and delivering care [8]. Therefore, nurses’ competence for disaster management is a combination of knowledge, skills and behaviors required by nurses to prepare and respond to natural or man-made disasters [9].

By acquiring these competencies, nurses can play an important and critical role in disaster mitigation, preparedness, response and recovery to minimize the negative impacts of disasters [10, 11]. In recent years, nurses’ disaster competencies have improved, but their competencies remain low in some countries [1, 12]. The literature review has consistently emphasized the development and training of disaster-related nursing competencies [2, 13] and suggests that health organizations should train and improve nurses’ disaster competencies [14, 15]. New training programs and curricula should be designed in this area [16]. So that nurses are continuously trained, gain experience and update their competencies in disaster risk management [17, 18]. With the advancement of technology, there has been a growing interest in online education. Studies emphasized the importance and need of using online education for continuous education. These studies suggest that successful online training requires careful planning, including defining the purpose, identifying the audience, selecting appropriate content, and choosing delivery methods such as live or recorded sessions [19, 20]. Online training offers several potential benefits such as increasing access, reducing costs, providing flexibility in timing, and improving results. Therefore, online training can be a valuable tool for educational and training purposes, especially during crises such as the COVID-19 pandemic, and for nurses who often face a heavy workload [21].

An Iranian study reported low to moderate disaster competencies of nurses and concluded that current disaster risk management training for nurses was not standard in terms of quantity and quality [22, 23]. The researchers emphasized that the training and empowerment of nurses was necessary to best respond to the needs of patients during disasters [24]. Several studies have used online training to develop nurses’ various competencies and increase their knowledge, awareness, skills and satisfaction [25, 26]. For example, one study showed the effect of online training in disaster management on nurses’ knowledge and awareness and suggested that the effect of online training on nurses’ disaster practice and competency in different settings should be explored [16]. A review study examined the available training options for disaster response and focused on the effect of online training in combination with other methods such as scenarios, lectures, simulations and maneuvers. Above study also suggested that online training should be used for clinical and non-clinical aspects of disaster preparedness and response [24]. Given the importance of improving nurses’ disaster preparedness and competencies, further interventional studies are needed in this regard. This study aimed to determine investigating the effectiveness of an online training program to improve the competence of nurses in disaster risk management.

## Methods

### Study design and settings

This interventional study had a pretest-posttest control group design and the research setting was a specialized trauma hospital affiliated with Kerman University of Medical Sciences in southeastern Iran, in 2023.

### Population and sampling

The study population included all nurses working in specialized trauma hospital. The inclusion criteria were nurses with at least a bachelor’s degree, and one year of clinical work experience. The exclusion criteria were unwillingness to participate in the research and being on leave for more than two weeks during the last month. To calculate the sample size, we considered the study of Qazaljeh et al. [15], considering the standard deviations of 2.39 and 1.68 before and after the intervention, and the minimum mean difference of 1.5, the test power of 80%, the confidence level of 95%, type I error and type II error of α = 0.05 and β = 10%. The study sample was estimated to be 40 individuals for each of the intervention and control groups, but 42 were selected for certainty (84 in total). In the first step, we invited volunteer nurses with convenience sampling to participate in the training program through the nursing school website and the hospital supervisor training channel. In the second step, registered nurses were allocated randomly as the intervention (n = 42) and control groups (n = 42). Finally, 81 nurses completed the questionnaires and three nurses from the control group were excluded because they failed to fill in the posttest questionnaires completely (response rate = 96.42).

### Data collection and instrument

After receiving necessary permissions and coordination, the researcher created two intervention and control groups on WhatsApp. All participants gave their informed consent by completing an electronic form. Both groups completed anonymous online self-report questionnaires simultaneously before and after the intervention (one month later).

In this study two tools were used to collect data.

#### 1) Demographic information questionnaire

containing 8 items (age, sex, education level, professional experience, participation in disaster risk management training, ward, working position, employment status, prior disaster experience, marital status. We also asked three questions about nurses’ awareness of their roles before, during, and after disasters by yes and no.

#### 2) The Nurses’ Perceptions of Disaster Core Competencies Scale (NPDCC)

Celik (2010) developed, measured psychometrically, and validated NPDCC in Turkey [27]. This scale includes 45 items and 5 subscales: critical thinking skills (4 items), special diagnostic skills (6 items), general diagnostic skills (13 items), technical skills (14 items), and communication skills (8 items). The scores were rated on a five-point Likert scale, with one corresponding to “this should be trained” and 5 corresponding to “I can do and train. The minimum score was 45 while the maximum score was 225, with a higher score indicating nurses’ better perceptions of disasters core competencies [28]. To facilitate comparison of scores with other studies, we considered a score of 45–105 as weak competency, a score of 105–165 as moderate competency, and a score above 165 as high competencies.

The researchers confirmed original NPDCC reliability using internal consistency and reported Cronbach’s alpha coefficient of 0.81–0.92 and 0.96 for the subscales and the whole scale, respectively [28]. After obtaining permission, we used translation-back translation method to culturally adapt the Persian version of the questionnaire; the research team translated the questionnaire into Persian, two English language experts translated it into English. The number of inconsistencies was then checked and the Persian version of the questionnaire was prepared with changes in the item wording. To qualitatively determine the validity content, we gave the scale to 10 professors of nursing and midwifery school and the Health in Disasters Research Center and prepared the final scale after collecting their opinions. We gave this scale to 20 nurses to check their perceptions, but we made no changes in the questionnaire. The scale reliability was determined using a pilot study on 30 people. Cronbach’s alpha coefficients for critical thinking skills, special diagnostic skills, general diagnostic skills, technical skills, communication skills, and the whole scale were 0.86, 0.86, 0.94, 0.94, 0.96, and 0.96, respectively.

### Educational intervention

First, the researcher provided information about the process and timing of the educational intervention through the WhatsApp group. The intervention group received four 2-hour sessions from 9 to 11 am on Sundays and Tuesdays for two weeks. Two experts in the field of health in disasters and incidents approved educational materials aimed at empowering and improving nurses’ disaster competencies (Table 1). We used Adobe Connect platform to implement the online training program in the forms of lectures, group discussions, scenario presentations, case reports, questions and answers. Two research members, a faculty member from Kerman University of Medical Sciences and two PhD of health in disasters and incidents chaired the sessions. Educational files, including operational videos, scenarios, and educational audio and text files were provided to the participants through the WhatsApp group. The nurses were able to review the training materials and discuss their probable questions on the research team through WhatsApp. Some questions were also administered to the participants to answer and upload the WhatsApp group. The researchers provided nurses with feedback on their answers using WhatsApp. Reminders were sent to them on WhatsApp and they were asked to give feedback on the researchers.

The researchers reminded the intervention group not to share the educational content with the control group until the end of the study. To ensure accurate tracking of attendance, we used registration forms and attendance lists. As attendance is crucial to fully benefit from the training course and to evaluate its impact on the research results, we carefully monitored participants’ attendance. The training sessions were scheduled at times when participants were not required to perform work-related tasks. This was done to minimize distractions and interruptions, allowing participants to focus on the educational content.

**Table 1:** Summery of content of the Disaster Risk Management training program

Sessions		Main topics	Contents	
1	Principles and fundamentals of disaster risk management			- Definition and explanation of common terms in disaster risk management - Description of the components of the disaster risk management cycle - The concept of disaster mitigation - Explanation of the functions of disaster mitigation and management programs in different stages of the cycle - Familiarity with disaster mitigation and disaster preparedness - Familiarity with disaster response and disaster recovery
2	Familiarity with incident command system and Risk assessment and analysis			- Managerial changes when incidents and disasters occur in organizations - Communications in the incident command system chart - The duties of key people in the incident command system -Defining risk assessment - Introducing the elements related to the risk assessment process - Explaining the process of hospital risk assessment - Describing and explaining risk assessment tools
3	Healthcare services in disasters and psychosocial interventions in disasters			- The importance and necessity of how to provide healthcare services during disasters - Hospital evacuation and surge capacity, hospital incident command system -Evaluation of the injured in mass casualty incidents - Nursing management and care of trauma victims caused by mass casualty incidents - the concepts of mental health and psychosocial support team - the different stages of psychological reactions of people in disasters - psychological interventions for vulnerable groups in disasters - the symptoms of mental disorders in the victims - Standard psychosocial interventions in the affected area
4	Nursing in disasters and incidents, Triage in disasters			- The position and roles of nurses in disasters - The importance and necessity of nursing management in disasters - The duties of nurses based on special, technical and general competencies in disasters - The injured triage in disasters and incidents with the START method - Comparison of types of triages with START triage

### Statistical analysis

Data were analyzed using SPSS21 and descriptive statistics (frequency, percentage, mean and standard deviation). The Kolmogorov-Smirnov test was used to assess the normality of the data, and then an independent samples t-test was conducted to compare the NPDCC scores between the two groups before and after the intervention. Additionally, a paired t-test was used to compare the NPDCC scores within each group before and after the intervention. To control the impact of pretest scores on NPDCC scores, an analysis of covariance was applied. A significance level of 0.05 was considered.

## Results

Nurses’ demographic and professional information Table 2 shows the participants’ demographic and professional information for the intervention and control groups. A total of 81 nurses completed the study and returned the questionnaires (response rate 96.42%). The two study groups were homogenous in terms of demographics and professional information except for gender. Mean age was at the intervention group (39.45 ± 6.53) and at control group (36.69 ± 7.99). Most of the nurses in the intervention and control groups were women (90.5, 59%), married (88.1, 82.1%), clinical nurses (78.6, 74.5%), undergraduate education (76.2, 84.6%) with 11–20 years of work experience (71.4, 48.7%), had permanent employment (69, 56.4%). In addition, they mostly were nurses (26.2, 38.5%) working in the emergency department. No differences were found in the baseline measures of demographic variables except for gender.

**Table 2:** Comparison of demographic and professional information of the two study groups

Variables	Categories	Intervention	Control	Test	p-value
Gender	Female	38(90.5)	23(59)	χ^2^ = 10.79	0.001*
	male	4(9.5)	16(41)		
Professional experience (year)	1–5	2(4.8)	3(7.7)	χ^2^ = 7.58	0.10
	6–10	4(9.5)	9(23.1)		
	11–20	30(71.4)	19(48.7)		
	> 20	6(14.3)	5(12.8)		
Education level	Bachelor’s degree	32(76.2)	33(84.6)	χ^2^ = 2.57	0.27
	Master’s degree	10(23.8)	5(12.8)		
	PhD degree	0	1(2.6)		
Marital status	Married	37(88.1)	32(82.1)	χ^2^ = 0.65	0.72
	single	4(9.5)	6(15.4)		
	other	1(2.4)	1(2.6)		
Ward	ICU	11(26.2)	9(23.1)	χ^2^ = 9.92	0.12
	Emergency	11(26.2)	15(38.5)		
	Surgery	6(14.3)	4(10.3)		
	Operating room	5(11.9)	1(2.6)		
	Supervisors & Manager of nurse	0	1(2.6)		
	Pediatric	0	4(10.3)		
	Other	9(21.4)	5(12.8)		
history attending in disaster risk management trainings	Yes	26(61.9)	24(61.5)	χ^2^ = 0.001	0.97
	No	16(38.1)	15(38.5)		
Position	Nurse	39(92.9)	30(76.9)	χ^2^ = 5.07	0.16
	Head nurse	1(2.4)	4(10.3)		
	Supervisors	2(4.8)	3(7.7)		
	Others	0	2(5.1)		
Employment status	Committed	0	3(7.7)	χ^2^ = 3.89	0.14
	Temporary-to-permanent	13(31)	14(35.9)		
	Contract recruiters	29(69)	22(56.4)		
Awareness of nurses’ roles before the disaster	Yes	37(88.1)	33(84.6)	χ^2^ = 0.2	0.64
	No	5(11.9)	6(15.4)		
Awareness of nurses’ roles during the disaster	Yes	39(92.9)	32(82.1)	χ^2^ = 2.18	0.14
	No	3(7.1)	7(17.9)		
Awareness of nurses’ roles after the disaster	Yes	41(97.6)	39(100)	χ^2^ = 0.94	0.33
	No	1(2.4)	0		
		**Mean ± SD**	**Mean ± SD**	**Test**	**p-value**
Age		39.45 ± 6.53	36.69 ± 7.99	t = 1.7	0.09

*Bold p-value a is significant at the level of ≤ 0.05

Table 3 shows the level of competency of nurses in disaster risk management and its subscales for the two study groups before and after the intervention. The results of an independent t-test showed that the intervention and control groups were not significantly different in terms of their scores before the training. After the training, there was no significant difference in the total scores between the two groups. However, the intervention group showed a significant improvement in their scores for critical thinking, special diagnostic, and communication skills subscales after the training as compared to their scores before the intervention. The increase in scores for the technical skills and general diagnostic skills subscales was not statistically significant.

**Table 3:** Comparison of competency scores in the intervention and control groups based on NPDCC before and after the DRM training

Variable	Time	Prior to the intervention	After the intervention	Mean difference	ES^*^ (Cohen’s d)	Paired *t*-test	*P*- value
	Groups	M ± SD	M ± SD				
Critical thinking skills	Intervention	15.37 ± 3.11	16.19 ± 2.22	0.85	0.4	2.01	0.05*
	Control	16.30 ± 1.62	15.74 ± 1.74	0.56	0.33	-1.6	0.11
	Independent *t*- test	-1.66	1				
	*P*-value	0.10	0.32				
	ES^*^ (Cohen’s d)	0.37	0.22				
Special diagnostic skills	Intervention	20.8 ± 4.54	23.11 ± 2.83	2.4	0.61	3.52	0.001*
	Control	22.61 ± 3.57	22.02 ± 2.04	0.58	0.2	-1.16	0.25
	Independent *t*- test	-1.97	2				
	*P*-value	0.052	0.49				
	ES^*^ (Cohen’s d)	0.44	0.44				
General diagnostic skills	Intervention	50.32 ± 7.06	52.59 ± 5.22	2.2	0.36	1.81	0.07
	Control	52.53 ± 5.35	51.48 ± 4.26	1.05	0.21	-1.48	0.14
	Independent *t*- test	-1.56	1.04				
	*P*-value	0.12	0.3				
	ES^*^ (Cohen’s d)	0.35	0.23				
Technical skills	Intervention	57.6 ± 8.64	59.73 ± 6.33	1.85	0.21	1.31	0.19
	Control	58.02 ± 6.59	58.33 ± 5.8	0.30	0.04	1.94	0.7
	Independent *t*- test	-0.24	1.03				
	*P*-value	0.8	0.3				
	ES^*^ (Cohen’s d)	0.05	0.23				
Communication skills	Intervention	28.82 ± 6.24	31.69 ± 4.43	2.9	0.53	2.83	0.007^*^
	Control	30.10 ± 3.95	30.66 ± 3.65	0.56	0.14	0.91	0.36
	Independent *t*- test	1.51	15.30				
	*P*-value	0.134	0.001^*^				
	ES^*^ (Cohen’s d)	0.32	3.13				
Total of the NPDCC	Intervention	172.92 ± 26.27	183.33 ± 18.07	10.2	0.46	2.43	0.02^*^
	Control	179.58 ± 16.91	178.25 ± 13.71	1.33	0.08	-0.66	0.51
	Independent *t*- test	-1.33	1.41				
	*P*-value	0.18	0.16				
	ES^*^ (Cohen’s d)	0.3	0.31				

*Bold p-values are significant at the level of ≤ 0.05, Effect size (ES):0-0.2 = small effect, 0.2–0.5 = moderate effect, > 0.5–0.7 = large effect; and > 0.7 = very large effect

The analysis of covariance was used to investigate the effects of pre-test, gender and history attending in disaster risk management trainings on nurses’ scores in the NPDCC. The results showed a significant difference in the scores of the intervention group after training program, suggesting that the training program had a positive effect on nurses’ competence in disaster risk management, even after accounting for the effects of pre-test, gender and history attending in disaster risk management trainings (Table 4). These results are also consistent with the results of Table 3.

**Table 4:** Results of covariance analysis for the two groups of control and intervention

Dimensions		Type III sum of squares	Df	Mean square	F	P-value
Critical thinking skills	Intercept	166.97	1	166.97	48.84	0.001
	Pretest	4.88	1	4.88	6.05	0.27
	History attending in disaster risk management trainings	1.19	1	1.19	0.3	0.71
	Gender	2.11	1	2.11	0.61	0.43
	Group	14.45	1	14.45	4.22	0.04
	Error	256.36	75	3.41		
	Intercept	604.12	1	604.12	118.63	0.001
Special diagnostic skills	pretest	3.51	1	3.51	0.69	0.40
	History attending in disaster risk management trainings	11.17	1	11.17	1.17	0.57
	Gender	28.51	1	28.51	5.41	0.09
	Group	53.6	1	53.6	10.52	0.002
	Error	381.93	75	5.09		
	Intercept	1190.32	1	1190.32	60.34	0.001
General diagnostic skills	Pretest	51.44	1	51.44	2.6	0.11
	History attending in disaster risk management trainings	39.06	1	39.06	1.7	0.69
	Gender	26	1	26	3.38	0.21
	Group	89.43	1	89.43	4.53	0.03
	Error	1479.35	75	19.72		
	Intercept	1061.18	1	1061.18	40.15	0.001
Technical skills	Pretest	33.45	1	33.45	2.1	0.37
	History attending in disaster risk management trainings	11.9	1	11.9	1.62	0.86
	Gender	25.94	1	25.94	3.3	0.34
	Group	119.01	1	119.01	4.5	0.03
	Error	1981.93	75	26.42		
	Intercept	663.18	1	663.18	46	0.001
Communication skills	Pretest	12.81	1	12.81	1.37	0.11
	History attending in disaster risk management trainings	9.86	1	9.86	0.98	0.78
	Gender	54.12	1	54.12	3.75	0.06
	Group	72.99	1	72.99	5.06	0.02
	Error	1081.21	75	14.41		
	Intercept	12811.76	1	12811.76	62.76	0.001
Total of NPDCC	Pre test	42.77	1	42.77	2.20	0 1
	History attending in disaster risk management trainings	35.01	1	35.01	1.19	0.59
	Gender	10.99	1	10.99	1.36	0.2
	Group	1658.53	1	1658.53	8.12	0.006
	Error	15308.79	75	204.11		

Bold p-values are significant at level of ≤ 0.05

## Discussion

The findings of the study revealed that the online training program was effective in enhancing the total score and subscale scores for the intervention group. However, the increase in scores for the technical skills and general diagnostic skills subscales was not statistically significant. Xia et al. (2020) studied the impact of the disaster management training program on the preparedness and competencies of nursing students in China and found that the training program significantly improved disaster preparedness and competencies of nursing students [29]. One study in Turkey used a module to examine the impact of disaster management training on final year undergraduate nursing students and showed that the training significantly improved participants’ knowledge and attitudes towards disaster management [30]. An Iranian study reported that the training program improved nurses’ knowledge and attitude, disaster preparedness and response [18].

The study results showed no significant difference in the scores of disaster competencies between the two groups after the intervention. Kim et al. (2014) studied the effect of an online training on the sexual health care competencies of nurses in Korea and demonstrated that the educational intervention could not significantly improve nurses’ attitude and practice scores in the intervention group compared with the control group [31]. Another study investigated the effect of online training on the clinical competencies, critical thinking and problem solving of nurses and found no statistically significant difference in the problem-solving and critical thinking skills between the two groups after the training program [32]. There could be various reasons for the absence of a significant difference between the two groups. One possible explanation is that the study had limitations in terms of time and facilities. Nurses may have lacked motivation and time to attend training sessions due to their heavy workload and not perceiving the need for such training. Additionally, based on the experiences of nurses in previous studies, the development of disaster competencies in nurses requires both theoretical and practical training. Although theoretical classes can enhance nurses’ knowledge, practical training is also essential [29]. Soo-huh et al. (2019) used video, lecture, and group discussion to train disaster competencies in practice and theory and showed a significant increase in the mean scores of disaster preparedness, competencies and attitudes of nurses in the intervention group compared to the control group after the intervention [33]. The results of their study are different from current studies. They performed nurses’ educational need assessment before designing the training program and used several educational approaches to implement the training program, which could increase the effectiveness of the training. In another study, nurses received disaster risk management training through web-based simulation approaches and reported higher levels of knowledge and perceived competencies. Researchers emphasized that various educational methods and approaches could increase the effect of disaster management training on nurses [22].

Another study in Turkey provided educational videos and presentations in the form of modules using the LMS (Learning Management System) and showed that the training program could increase the mean scores of perception and self-efficacy of nurses in the intervention group compared with the control group and affected nurses’ disaster preparedness [34]. Asynchronous presentation of educational materials, discussion of the educational content and student-teacher interaction could encourage learning LMS reminded individuals to study the modules, and if they failed one module, it was impossible to see other modules. Sattar et al. (2019) used educational content and approaches similar to current study, but the training program was in person with more sessions. They showed that the educational intervention could increase the disaster-related knowledge, attitudes and preparations of nurses in the intervention group compared with the control group [35]. However, a review study reported that the effect size of online training could be different depending on the skills trained, the training outcome and the educational approach used, and nurses needed continuous practical exercises, maneuvers and simulated situations for specialized skills, such as disaster management competencies because they experienced them in special and limited situations [36].

The study results showed no difference in the technical skills subscale of nurses before and after the intervention. Technical skills are not new for nurses, although they have increased dramatically as a result of nurses’ involvement in COVID-19 and their interest in participating in disaster-related training. According to one study, nurses received limited and insufficient training in communication skills, but training programs could effectively improve the communication skills of nurses [37].

### Limitations

This study had limitations that are taken into account when interpreting the findings. First, given that the participants are selected from a specialized trauma hospital by a convenience sampling, the generalizability of the findings should be performed with caution. Second, the majority of nurses (61%) in the control and intervention groups had a history of participating in disaster management training courses. Due to the limited sample size, we could not exclude them from the study. On the other hand, probably most of the nurses participated in in-service training regarding for disaster risk management during the covid-19 pandemic or received advanced training during their studies at the university. Therefore, this might have affected results. This limitation was controlled using covariance analysis. Third, we used a self-report instrument to assess nurses’ disaster core competencies. Therefore, the evaluation is done at the elementary levels of the Kirkpatrick model, such as the reaction level. Fourth, due to the limitation of virtual training infrastructures in hospitals we only used Adobe Connect platform to implement the online training and could not use blended methods and other electronic training methods such as Learning Management Systems (LMS). Moreover, the study did not include practical exercises, maneuvers, and simulated situations, which are crucial to developing specialized disaster management competencies. Finally, we suggest for future research to evaluate the effectiveness of educational programs using more complete blended teaching methods such as LMS, practical exercises, maneuvers, and simulated situations at higher levels of the Kirkpatrick model such as behaviors, outcomes and consequences [38]. Evaluation of disaster core competencies is strengthened through a combination of different approaches such as of Kirkpatrick’s four-level model and 360-degree method with controlling confounding variables and future longer follow-ups.

## Conclusion

The study findings suggest that online training can significantly enhance the NPDCC score of nurses in the intervention group; although the increase was not statistically significant when compared to the control group. To optimize the benefits of virtual education, it is recommended to combine it with other teaching methods to promote interaction and create a collaborative learning environment, which can lead to more effective interventions. Incorporating practical exercises and maneuvers can help nurses gain hands-on experience and improve their perception of the core competencies required for better disaster management.

## Data Availability

The data are available upon request to the corresponding author after signing appropriate documents in line with ethical applications and the decision of the ethics committee.

## Abbreviations

NPDCC: Nurses’ perceptions of disaster core competencies scale
LMS: Learning Management System
